# In Memoriam: Francisco Javier Lopera Restrepo

**DOI:** 10.1590/1980-5764-DN-2024-IM01

**Published:** 2025-01-17

**Authors:** Ricardo Nitrini

**Affiliations:** 1 Universidade de São Paulo, Faculdade de Medicina, Hospital das Clínicas, Departamento de Neurologia, Grupo de Neurologia Cognitiva e do Comportamento, São Paulo SP, Brazil.

Francisco Javier Lopera Restrepo passed away on September 10, 2024, a great loss for his family, friends, and collaborators, for all of science, and, particularly, for neurosciences in Latin America. He leaves the advances brought by his studies as well as a great legacy that will certainly have important developments to be carried out by the large number of high-quality disciples he left behind.

Much will be written about the importance of Francisco Lopera for advancing knowledge about Alzheimer's disease. There are aspects that may be considered more important for the entire research community, especially for those from low- and middle-income countries.

Almost all Francisco Lopera's publications are about autosomal-dominant Alzheimer's disease, which was described in 1987^1^. Collaboration with a large group of investigators led to the discovery of the E280A mutation of presenilin 1 in 1995^2^. From his careful studies of this family, he was able to obtain enough data to transform it into the world's largest autosomal-dominant Alzheimer's disease kindred, which made possible to make contributions to the knowledge of several steps of the evolution of Alzheimer's disease.

Two of them are highlighted here. The first one made it possible to verify the first changes in biomarkers, the time interval between these changes, and the first symptoms hallmarking the onset of dementia. These discoveries revealed that the interval between changes in biomarkers and dementia is greater than 20 years^3,4^.

**Figure:**
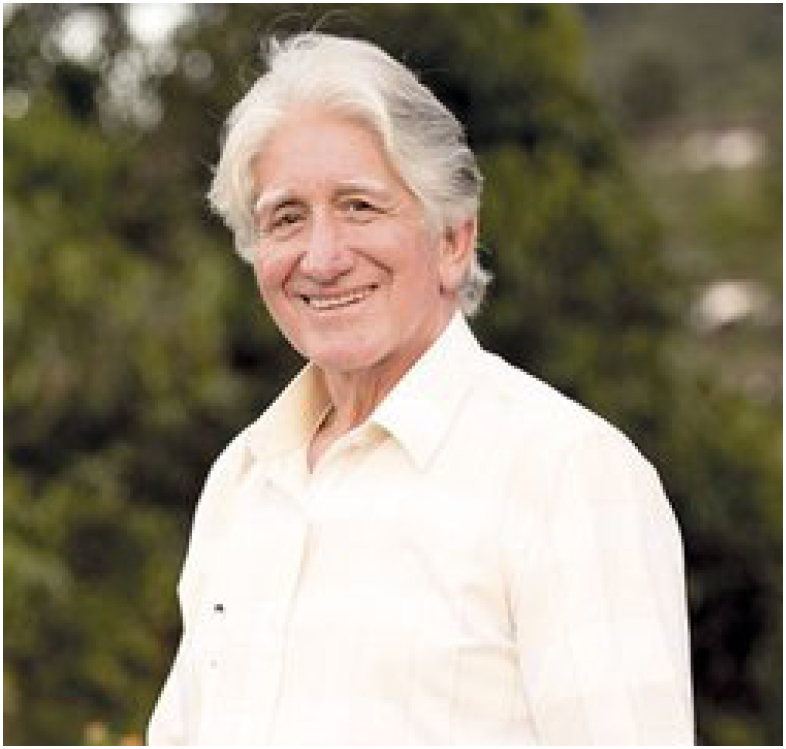


The second one is the case of the patient Aliria, who, having the E280A mutation of presenilin 1, did not develop mild cognitive impairment until her seventies, three decades after the expected age of clinical onset^5^. Aliria died at the age of 77 years without dementia, when the average age at which dementia occurs in the disease caused by this mutation is about 49 years^5,6^. The neuropathological examination confirmed the presence of unusually high brain amyloid levels and limited tau and neurodegenerative measurements^6^, which had already been revealed by amyloid-positron emission tomography (PET) and tau-PET performed years before^5^. The patient had two copies of the APOE3 Christchurch (R136S) mutation, which points to the role of APOE in the pathogenesis, treatment, and prevention of Alzheimer's disease^5,6^.

These and many of his other contributions were rightly awarded, most notably the Bengt Winblad Lifetime Achievement Award in 2020, from the Alzheimer's Association, and the Potamkin Prize (often called the "Nobel Prize of Alzheimer's research"), from the American Academy of Neurology (AAN) and the American Brain Foundation (ABF), in 2024.

In addition to the importance of these studies for scientific knowledge, there is much to be learned from their studies by all researchers in Latin America.

First, tenacity, persistence, maintaining focus, and the firm conviction that this determination would bring more and more important discoveries. Between the first publication on this disease and the Aliria case, 32 years of continued studies passed.

Second, but no less important, he had good collaborators among his colleagues and students and chose the best international collaborators, in whom he could trust and who did not look at a researcher from a low- and middle-income country as a mere data provider to enrich their own research. Despite the depth of his research that he would not have achieved if he had not had international collaboration, Francisco Lopera always remained the main researcher and unquestionable leader of his publications.

According to Natalia Acosta-Baena, one of his closest collaborators, he was always concerned with improving the quality of life of the patients who were being under study by his group, and he kept "a balance between consent without falling into coercion" while dealing with this vulnerable population.

Like many neurologists of his time, Francisco Lopera began studying dementia through studies in neuropsychology, as can be seen from some articles on these topics among his first publications^7,8^, and during his career, he had kept an in-depth knowledge of cognitive and behavioral neurology.

Francisco Lopera was a member of the Advisory Board of Dementia & Neuropsychologia, and together with his group, he published a paper entitled "Genetics of dementia: insights from Latin America^9^," a relevant contribution to the field and to our journal.

Finally, Francisco Lopera could have been nominated for the Nobel Prize, which would have been another of his formidable examples for the entire community of neuroscientists in Latin America and abroad.
